# The hospital of tomorrow in 10 points

**DOI:** 10.1186/s13054-017-1664-7

**Published:** 2017-04-11

**Authors:** Jean-Louis Vincent, Jacques Creteur

**Affiliations:** Department of Intensive Care, Erasme Hospital, Université libre de Bruxelles, Route de Lennik 808, 1070 Brussels, Belgium

## Abstract

Technology has advanced rapidly in recent years and is continuing to do so, with associated changes in multiple areas, including hospital structure and function. Here we describe in 10 points our vision of some of the ways in which we see our hospitals, particularly those in developed countries, evolving in the future, including increased specialization, greater use of telemedicine and robots, the changing place of the intensive care unit, improved pre-hospital and post-hospital management, and improved end-of-life care. New technology is going to increasingly impact how we practice medicine. We must learn how best to adapt to and encompass these changes if we are to achieve maximum benefit from them for ourselves and our patients. Importantly, while the future hospital will be more advanced technologically, it will also be more advanced on a personal, humane patient care level.

## Background

The speed with which technology and clinical informatics is advancing is truly amazing and with it come associated changes in multiple areas that are increasingly impacting on everyday life. Our hospitals are no exception to this rule and the hospital of the future is going to be very different from the hospital of today. Here we provide a personal view of 10 areas we believe we will see important changes in hospital design and function in the not so distant future. Clearly our predictions are subjective and there will be many aspects we have not covered; nevertheless, we believe that our future hospitals will look something like this.

## 1. Hospitals will be smaller and more specialized

There will be fewer hospital beds overall than is currently the case, for multiple reasons, including: greater focus on, and more effective, disease prevention; more rapid patient work-ups (imaging and laboratory), diagnostic tests, and treatments leading to shorter hospital stays; wider use of non-invasive interventions enabling shorter convalescence times; and vastly improved outpatient and home management. Primary care centers will be better staffed and equipped, enabling many more conditions to be diagnosed and managed without hospital admission. Hospitalization will be reserved almost exclusively for patients with severe acute illness. Moreover, the “general” hospital will gradually cease to exist because improved diagnostics will enable patients to be referred immediately to the specialty hospital that best suits their diagnosis. More ambulances will be medically equipped, enabling a team of trained paramedics and/or physicians to diagnose and stabilize patients during transfer.

## 2. Hospitals will be more user-friendly

Hospitals will look more like four-star or five-star hotels than hospitals, with large admission/reception areas, shops and restaurants, and landscaped gardens where patients and relatives can sit or walk (weather permitting of course). There will be no restricted visiting hours, with relatives (including children) free to visit at all times and to remain present during interventions should they and the patient wish it. Parents will be encouraged to stay with their sick children and pediatric rooms will be equipped accordingly, with showers and kitchen areas to prepare meals. Hospital rooms will be light and spacious, and equipped with large interactive screens on which patients can see their own results and progress, request a consultation with their doctor or therapist by videolink, check out individualized medical information about their condition via Internet searches (see later), order room service, and connect online with other patients with similar conditions should they wish to. Patients will be better informed and patient autonomy will play a greater role in decision-making about all aspects of their care including treatment options and end-of-life care. The traditional one-size-fits-all concept of medical care will be replaced by a much more personal approach to patient management.

## 3. Staff numbers will be reduced

Much of the routine hospital administration (e.g., on admission and discharge) will be conducted via touchscreens (as is already the case in the Samsung Medical Center in Seoul, South Korea, for example). Electronic medical records will be updated automatically every time a test is ordered and results available immediately to all involved, including the patient. Sophisticated software will continuously integrate a patient’s signs and symptoms with the results and evolution of monitored variables and results of laboratory tests, propose and even initiate a treatment, and then monitor its effects [1], so that many aspects of management will be on “automatic pilot”. As a result, fewer doctors will be routinely present on the hospital floor; a skeleton staff will of course still be present to take care of emergencies and participate in the rescue or code blue teams on the floor.

Although there will be fewer staff, patient care will not be neglected. Nursing assistants will be responsible for routine aspects of patient management. Relieved of much of their time currently “wasted” on administration, routine tasks, and traveling between wards and departments, doctors and nurses will have more time to interact and communicate with patients and their families. Discussions will be informed by computer programs able to individualize each patient’s situation. All of the components/variables, including trend analyses, relevant to the individual patient will be analyzed and presented graphically so that the patient and family will be able to better understand the issues involved in their case. These data will be linked to an appropriate website program—when a new diagnosis is made, instead of patients and relatives randomly “googling” the condition and being sent to multiple websites of dubious accuracy or relevance, the computer will direct them to scientifically verified information relevant to their specific case; for example, a patient with breast cancer does not need to read everything about all types of breast cancer, but rather just about the type and stage they have, thus providing a more accurate individual perspective.

## 4. Telemedicine will be everywhere

The technology for telemedicine is already available and its use is limited today largely by questions related to how best to apply it. Image quality and speed of transmission still need to improve, but already many hospitals worldwide use teleconsultations where local specialists are not available. This approach works particularly well for dermatological conditions where a photograph or video can be transferred rapidly to a specialist who can assist in diagnosis and provide therapeutic guidance. Radiology for interpretation of imaging studies and cardiology for analysis of complex cardiac rhythms are other areas that currently lend themselves easily to telemedicine, but the possibilities for telemedicine in the future are almost endless.

Augmented and virtual reality techniques are already used widely to enhance surgical technique and outcomes [2]. Remote telesurgery will also increasingly be used to perform surgical interventions, with surgeons operating from their office using remote robotic arms with no need to actually visit the hospitals in which they “practice” [3, 4]!

## 5. Robots will be more present and visible

Why do we need people to deliver food, linen, drugs, and so forth, to patient rooms? In a number of hospitals (e.g., Mission Bay Medical Center, University of California San Francisco, USA) these tasks are already done by robots. Using their own programmed elevators, food and other supplies can be brought automatically from one part of the hospital to another and even to the patient’s room. Robot “porters” will also be used to move patients around the hospital for different tests or interventions and robots will largely replace physiotherapists for exercise [5]. Importantly, the robots of the future will be much more lifelike than we can currently imagine and also able to converse and provide company or entertainment.

## 6. Improved non-invasive monitoring

On admission, patients will be fitted with a number of non-invasive multimodal probes or sensors that will continuously assess not only their heart rate and the oxygen saturation (by pulse oximetry), but also arterial pressure, temperature, respiratory rate, adequacy of skin perfusion, blood glucose levels, and so on [1]. Fluid balance will also be routinely recorded continuously. These data will be transmitted to and continuously monitored by a central console (in the hospital or elsewhere), which will alert a small team to check on the patient if necessary [6, 7].

## 7. Will there still be an ICU?

This is a difficult question, without a precise answer. One possibility is that there will be a department of intensive care (of course, there is no need for separate medical/surgical/trauma ICUs [8], although ICUs may become more “subspecialized” by the nature of the patients admitted to specialist hospitals). If such a department still exists, it will be very different from its current format [9]. Some experts suggest that rather than having a separate ICU, if a patient needs intensive care their regular hospital bed will simply be transformed, by bringing in a respirator and more sophisticated monitoring equipment without needing to transfer the patient. The arguments for and against these two suggestions are presented in Table 1. These choices may depend, at least in part, on the hospital and the particular problem faced by the patient. Because hospitalized patients of the future are likely to be more severely ill, the number of intensive care beds will increase whatever their physical placement within the hospital.

**Table 1:** Advantages and disadvantages of transforming ward beds into ICU beds

**Advantages**
• Avoids the hassle/additional workload of transfer procedures • Necessary equipment readily available (stored in bedside cupboards) but not obviously visible so less alarming for patients and relatives than an ICU environment • Virtually unlimited number of potential ICU beds (no “full ICU”) • Better continuity of care—no need for handover to another health care team • Avoids psychological obstacles to ICU transfer • No major step-up/step-down from one location to another
**Disadvantages**
• Difficulties having all of the sophisticated material readily available • Lack of professional expertise on the regular floor • Difficulties training the entire hospital (equipment, procedures, continuing education) • Need for intensivists to cross the hospital when needed for emergency intervention • Need for flexible staffing to face increased needs • Less exposure to and therefore experience of staff with complex end-of-life decisions

## 8. The patient will be mobilized early

The hospital bed will essentially be there for sleeping at night; whenever possible, patients will be ambulated early, often with the help of robots [5]. Of course, some patients will have to remain in bed, but even these patients will be helped to exercise passively and encouraged to exercise actively whenever possible (e.g., using programmed cycle ergometry) [10]. As already indicated, hospitals will be more user-friendly, encouraging patients to walk around and not to stay in their rooms. Thanks to their admission “smart” bracelet, hospital personnel will know exactly where the patient is at any time (e.g., in a specialty area for a test, in the cafeteria, the shopping mall, or outside in the garden) and will be alerted to any change in status from the continuous feedback of the monitoring probes.

## 9. There will be continuity between hospital and home care

Thanks to telemedicine, discharged patients will be managed “remotely” by the same team as in the hospital. With a large screen and a webcam, and continued non-invasive monitoring if required, the patient will be able to complete regular follow-up checks with a nurse and/or doctor via videocall, and to discuss any acute problems or concerns. The quality of the images and speed of connection will be such that it will be little different from being physically present in the hospital. This improved follow-up system will reduce the number of missed out-patient appointments post discharge and decrease readmission rates. Obviously, if dressings need to be changed or other procedures require a professional intervention, a mobile team can easily be dispatched to the patient’s home or the patient can attend the local primary care center.

## 10. Ethical decisions will be openly discussed and end-of-life care improved

There will be much more open discussion about end-of-life choices in and out of hospital and patients will have their wishes recorded early, preferably through a specially trained person (better than writing advance directives). End-of-life discussions and decisions will be facilitated by access to more accurate prognostic and quality-of-life data derived from continually updated analysis of big and deep data using sophisticated statistical programs. In patients whose condition will inevitably lead to death and in whom further treatment will not be of benefit, the end-of-life process can be started, aided by earlier open discussions and known, documented preferences. There will be greater acceptance of physician-assisted suicide and euthanasia around the globe [11], and the use of increasing doses of sedative agents with the sole purpose of shortening the end-of-life process and permitting death with dignity will be practiced, as it is already in Belgium [12], more widely.

## Conclusion

We have discussed 10 features of the future hospital as we see it, but we are sure there are many other aspects that we have not covered or that may conflict with others’ views—it is difficult to predict the future with any accuracy and many of these factors are subjective. The time scale for these changes is also impossible to predict and there will inevitably be a period of transition as the old and new mix. What is certain is that this whole area is going to evolve much faster than we can imagine. The key challenge will lie not only in technological developments, a number of which are already available (e.g., telemedicine and robots), but in how we implement and apply the new material. Determining who will be responsible for overseeing the technology, and who will really monitor patients in the ambulance, on the floors, or at home, will also be an important consideration. Although finance will be important, especially initially, costs are likely to decrease as these techniques are used more widely (as, for example, with new biological tests) and competition between brands increases. Importantly, while the future hospital will certainly be more advanced technologically, it will also be more advanced on a personal, humane patient care level.

New technology is going to increasingly impact how we practice medicine and cannot be ignored. We must learn how to adapt and encompass these new techniques if we are to achieve maximum benefit from them for ourselves and our patients.

## Abbreviations

ICU: intensive care unit
